# Effect of upper respiratory infection on anaesthesia induced atelectasis in paediatric patients

**DOI:** 10.1038/s41598-021-85378-0

**Published:** 2021-03-16

**Authors:** Hye-Mi Lee, Hyo-Jin Byon, Namo Kim, Stephen J. Gleich, Randall P. Flick, Jeong-Rim Lee

**Affiliations:** 1 Department of Anaesthesiology and Pain Medicine, Anaesthesia and Pain Research Institute, Yonsei University College of Medicine, 50-1 Yonsei-ro, Seodaemun-gu, Seoul, 03722 Republic of Korea; 2 Department of Anaesthesiology and Perioperative Medicine, Mayo Clinic, 200 1st SW, Rochester, MN 55905 USA

**Keywords:** Paediatric research, Respiratory tract diseases, Respiratory signs and symptoms

## Abstract

Upper respiratory tract infection (URI) symptoms are known to increase perioperative respiratory adverse events (PRAEs) in children undergoing general anaesthesia. General anaesthesia per se also induces atelectasis, which may worsen with URIs and yield detrimental outcomes. However, the influence of URI symptoms on anaesthesia-induced atelectasis in children has not been investigated. This study aimed to demonstrate whether current URI symptoms induce aggravation of perioperative atelectasis in children. Overall, 270 children aged 6 months to 6 years undergoing surgery were prospectively recruited. URI severity was scored using a questionnaire and the degree of atelectasis was defined by sonographic findings showing juxtapleural consolidation and B-lines. The correlation between severity of URI and degree of atelectasis was analysed by multiple linear regression. Overall, 256 children were finally analysed. Most children had only one or two mild symptoms of URI, which were not associated with the atelectasis score across the entire cohort. However, PRAE occurrences showed significant correspondence with the URI severity (odds ratio 1.36, 95% confidence interval 1.10–1.67, p = 0.004). In conclusion, mild URI symptoms did not exacerbate anaesthesia-induced atelectasis, though the presence and severity of URI were correlated with PRAEs in children.

*Trial registration:* Clinicaltrials.gov (NCT03355547).

## Introduction

A large number of children with upper respiratory tract infection (URI) undergo general anaesthesia for elective or emergency surgery even though URIs are known to increase perioperative adverse respiratory events (PRAEs)^1,2^. Meanwhile, patients of all ages who undergo general anaesthesia suffer anaesthesia-induced atelectasis which is one of the most pervasive adverse outcomes. In children, the incidence of atelectasis has been shown to range from 60 to 80%^3,4^.

A series of case studies and one prospective observational study demonstrated that severe pulmonary collapse occurred during anaesthesia in children who had preoperative URI symptoms which were not severe enough to postpone their surgery^5–7^. In addition, transient hypoxia after anaesthesia was observed in children with URI^8^. Accordingly, it is conceivable that anaesthesia-induced atelectasis is possibly aggravated and results in hypoxemia in children with URI^6,7,9,10^. However, no previous study has attempted to demonstrate a direct correlation between the severity of URI and the extent of anaesthesia-induced atelectasis.

Accurate knowledge on the development of atelectasis in children with URI is essential for optimal perioperative anaesthetic care. This prospective observational study aimed to investigate if the severity of URI symptoms is correlated with exacerbation of perioperative atelectasis in children.

## Methods

### Trial design and participants

Ethical approval for this study (IRB #4-2017-0766) was obtained from the Institutional Review Board of Severance Hospital, Seoul, Republic of Korea on 29th September 2017. All study methods were performed in accordance with the relevant guidelines and regulations. Written informed consent was obtained from the parents of all children. The trial was registered prior to patient enrolment at clinicaltrials.gov (NCT03355547, Principal investigator: Jeong-Rim Lee, Date of registration: 28th November 2017).

This study was designed as a prospective observational study of children aged 6 months to 6 years undergoing urologic, lower abdominal, or superficial general surgery under general anaesthesia. This study was conducted from 30th November 2017 to 1st February 2019. Patients were excluded if they underwent laparoscopic surgical procedures, were diagnosed with pneumonia or bronchiolitis at the time of surgery, or had a history of prematurity (< 37 weeks) or bronchopulmonary dysplasia. In addition, children with high fever (body temperature > 38.8 °C), abnormal lung sounds, or aggravated general weakness were excluded, and their surgery was postponed.

### Anaesthesia protocol

Intravenous cannulation was performed preoperatively in the ward per our hospital policy. When a patient arrived at an operating room, pulse oximetric readings and the electrocardiogram were monitored, and blood pressure was measured every 2.5–5 min. General anaesthesia was induced with propofol 2–3 mg kg^−1^, fentanyl 1–2 mcg kg^−1^, and rocuronium 0.6 mg kg^−1^. Mask ventilation was performed with 6 L min^−1^ of 100% O_2_ with 2.5–3 vol% of sevoflurane, and tidal volume was adjusted to about 8 mL kg^−1^ with peak inspiratory pressure of less than 15 cmH_2_O if possible. Tracheal intubation or supraglottic airway device (SAD) insertion was performed at least 3 min after rocuronium administration. After securing the airway, FiO_2_ was reduced to 0.5 and alveolar recruitment was performed by increasing the positive pressure up to 30 cmH_2_O step-wise. Initial mechanical ventilation was commenced in the volume-targeted pressure-controlled mode to deliver a tidal volume of 8 mL kg^−1^ at an inspiration:expiration ratio of 1:2 with zero positive-end expiratory pressure. The respiratory rate was adjusted to target an end-tidal carbon dioxide level of 35 to 40 mmHg. Anaesthesia was maintained with sevoflurane or desflurane at 0.8 – 1.0 minimum alveolar concentration. All anaesthetic management procedures were conducted by a paediatric anaesthesia specialist.

At the end of the surgical procedures, appropriate methods of analgesia were provided depending on surgery, volatile anaesthetic was discontinued, and atropine and neostigmine were administered for the reversal from residual muscle relaxation. Extubation was performed when the patient presented signs of grimace, eye opening, vocalisation, and spontaneous turning of the head. Then the patient was transferred to the post-anaesthesia recovery care unit (PACU), where standard monitoring was applied and vital signs were checked by nurses. The patient was observed for at least 30 min and discharged from the PACU when the modified Aldrete score was 9 or higher^11^.

### Survey of upper respiratory infection symptoms

To evaluate the presence and severity of URI, the ‘URI score’ was obtained from a modified existing questionnaire^12^ completed by the primary caregiver before anaesthetic induction. This questionnaire assessed 8 URI symptoms and each item is scored from 0 to 3 points; thus, the severity of URI is presented as a sum of the scores, from 0 to 24 points (Table 1). In addition, information on the duration of symptoms, whether any cold medication was taken, parents’ smoking history, and the child’s history of asthma or allergy were also gathered.

**Table 1:** Questionnaire filled by the caregivers rating the presence and severity of upper respiratory infection (URI) symptoms in children^12^.

Symptom	0	1	2	3
Sneezing	No sneezes	Few short episodes of sneezing	Occasional sneezing	Frequent sneezing
Runny Nose	No runny nose	Had to wipe (or blow) nose rarely	Had to wipe (or blow) nose occasionally	Had to wipe (or blow) nose frequently
Nasal congestion	No congestion	Slight breathing through nose	Noisy breathing through nose, has “nasal” speech, breathes through mouth sometimes	Breathes through mouth almost all the time because of nasal congestion, speech very “nasal”
Cough	No cough	Few short episodes of coughing	Occasional coughs or rare episodes of prolonged coughing	Frequent coughs or at least occasional episodes of prolonged coughing
Feverishness	No fever or flushed appearance	Felt warm to the touch, no flushing	Felt very warm to the touch or temperature > 38.0 °C, slightly flushed	Felt hot to the touch or temperature > 38.8 °C, very flushed
Chillness	No chillness	Complaining about being cold, no extra clothing or blankets	Wearing extra clothes or using a blanket to keep warm	Very chilled, shivering, constantly under a blanket to keep warm
Sore throat	No sore throat	Mild pain with swallowing	Moderate pain with swallowing	Very painful to swallow
Hoarseness	No change in voice	Speech is slightly hoarse or husky	Speech is very hoarse or husky	Can’t speak above a whisper because or hoarseness

The Questionnaire was obtained and modified from Taylor et al. *Pediatr. Res*. 2010 Sep;68(3):252–7.

### Lung ultrasonography and scoring of atelectasis

Transthoracic pulmonary ultrasonography was performed according to a previously published method^3^, which has been shown to be effective for diagnosing pulmonary atelectasis in children^13^. One designated researcher who was blinded to the child’s URI symptoms and experienced with sonographic measurement of atelectasis conducted the sonographic examinations.

**Figure 1:**
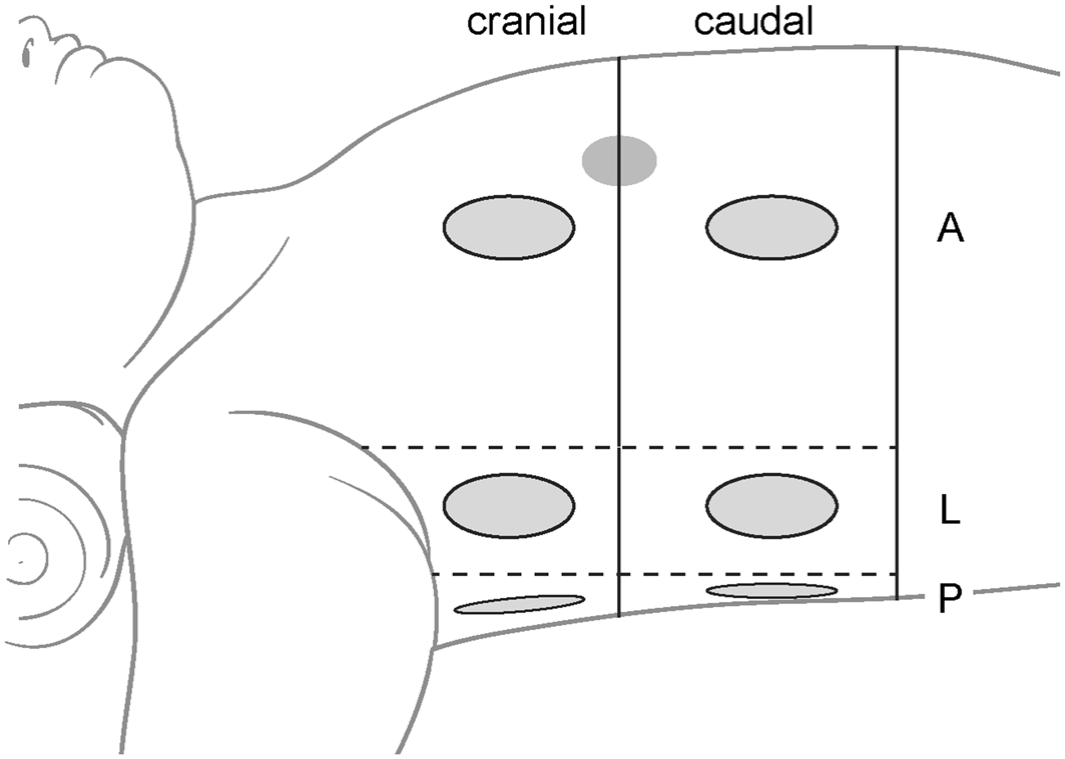
Segmentation of lung ultrasonography. The black axial line divides the thorax into the cranial and caudal regions based on the nipple. The vertical line divides the lung into A: anterior, L: lateral, P: posterior based on the parasternal, anterior, and posterior axillary line.

Pulmonary ultrasonography was performed twice in each patient. The first examination was conducted within 3 min of starting mechanical ventilation after induction of general anaesthesia. Lung recruitment was performed just after the first sonographic examination. The second examination was conducted at the end of the surgery. The 6–13-MHz linear probe of an ultrasonic device (LOGIQ-e, GE Healthcare, Wauwatosa, WI, USA) was applied vertically to the children’s ribs (anterior and lateral) or horizontally between the ribs (posterior), and a 2-dimensional classic view (depth 4 cm) was obtained. The segmentation for ultrasonography was divided into six regions per hemi-thorax, and 12 regions were evaluated overall (Fig. 1).

Atelectasis scoring was based on the findings of juxtapleural consolidation and B-line. The degree of juxtapleural consolidation was divided into four grades and scored between 0 and 3: 0, no consolidation; 1, minimal consolidation; 2, small-sized consolidation; and 3, large-sized consolidation. The degree of B-lines was also divided into four grades and scored between 0 and 3: 0, fewer than three isolated B-lines; 1, multiple well-defined B-lines; 2, multiple coalescent B-lines; and 3, white lung^3^. The ‘atelectasis score’ was presented as the sum of the scores of the 12 areas of consolidation and B-lines, from 0 to 36, respectively.

During recovery from anaesthesia, the other designated researcher who was blinded to the child’s URI symptoms recorded the occurrence of PRAEs, including laryngospasm, bronchospasm, sustained cough, or desaturation events (SpO_2_ < 95% on room air in PACU)^2^.

### Outcomes

The primary outcome was to define the correlation between URI and atelectasis scores after induction and at the end of the surgery. Secondary outcomes, including the correlation between other patient characteristics and atelectasis scores, were also analysed. In addition, the correlation between URI scores, patient characteristics, and PRAEs were analysed.

### Sample size calculation

As this study was an observational study, so any patients aged 6 months to 6 years who underwent general anaesthesia for paediatric general or urologic surgery from 30th November 30, 2017 to 1st February 1, 2019 were eligible in the study, except those who met any of the exclusion criteria.

### Statistical analysis

Statistical analysis was performed using SAS (version 9.4, SAS Inc., Cary, NC, USA). For assumption of normal distribution, Shapiro–Wilk test was used. On the basis of the normality of data, continuous variables (age, height, weight, duration of operation, duration of anaesthesia, URI score, URI onset day, and scores for pulmonary atelectasis) were expressed as mean numbers (SD). All categorical and ranking variables (sex, ASA PS [American society of anaesthesiologists physical status], type of surgery, airway device use, URI medication, and history of paternal smoking, asthma, and drug allergy) were expressed as number and percentage (%).

To determine whether the severity of URI was associated with the atelectasis score, multiple linear regression analysis was performed. The statistical result of this analysis was expressed as coefficient; ß and p value. The variables used in the regression analysis were analysed by factors that significantly increased PRAEs in general anaesthesia in children with URI symptoms in a previous study^1^. In the linear regression model, there was multicollinearity between the anaesthesia time and operation time (variance inflation factor ≥ 10), so the anaesthesia time was used for analysis. Missing data​ were excluded from the analysis.

The factors associated with PRAEs were analysed with univariate logistic regression. Since the number of events (n = 14) was too small to be modelled by one-in-ten rule, only univariate logistic regression was used. The results were expressed as odds ratio (OR), 95% confidence interval (CI), and p values. *P* < 0.05 was considered statistically significant.

## Results

**Figure 2:**
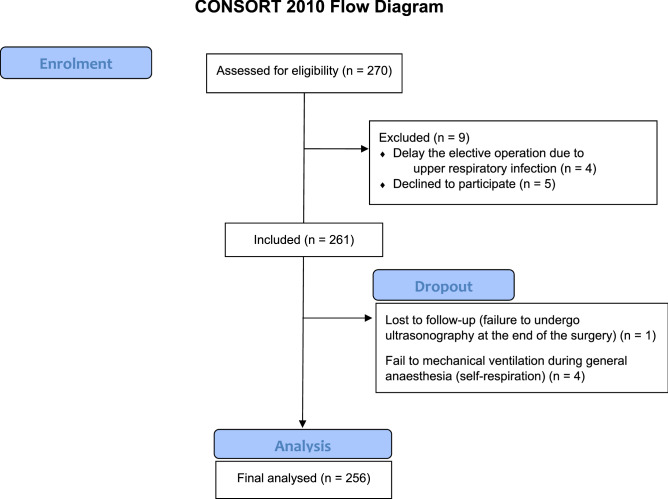
Flow diagram of the study.

Overall, 270 children were enrolled in the study. Of these, nine patients were excluded; three patients’ caregivers refused to participate, and elective surgery was postponed in six children due to severe URI symptoms. Of the remaining 261 patients, one patient was lost to follow-up because ultrasonography could not be performed at the end of surgery by the designated researcher due to conflicting schedules with another operation, and four children showed restoration of spontaneous respiration before the end of surgery. Therefore, 256 patients were finally analysed (Fig. 2).

Patient characteristics are presented in Table 2. The most common URI symptom was ‘runny nose’ (37.8%), and the symptom scores were just 1 or 2 in 49% of the children with URI.

**Table 2:** Characteristics of children included in the present study.

Variables		Mean (SD), Median (IQR [range]) or n (%)	
*General characteristics*			
Age (month)		16.0 (9.0–32.0 [6.0–72.0])	
Weight (Kg)		10.8 (9.2–13.7 [6.0–27.0])	
Height (cm)		79.1 (70.8–91.1 [51.0–117.5])	
Sex (M/F)		233 (91.0)/23 (9.0)	
Surgery (General/Urologic)		38 (14.8)/218 (85.2)	
General	Herniorrhaphy	21 (8.2)	
	Epidermal cyst excision	14 (5.5)	
	Sistrunk operation	3 (1.2)	
Urologic	Herniorrhaphy	71 (27.7)	
	Orchiopexy	67 (26.2)	
	Penoplasty	45 (17.6)	
	Repair of hypospadias	35 (13.7)	
Airway device (SAD/intubation)		221 (86.3)/35 (13.7)	
ASA PS (1/2/3)		154 (60.2)/101 (39.5)/1 (0.4)	
Operation time (min)		46.3 (34.4)	
Anaesthesia time (min)		75.0 (36.9)	
*URI-related information*			
		*No (n, %)*	*Yes (n, %)*
URI symptom		137 (53.5)	119 (46.5)
URI score^a^		n/a	1.4 (2.0)
URI onset day (day)^b^		n/a	3.0 (5.0)
URI medication		184 (71.9)	72 (28.1)
*Other medical/parental information*			
		*No (n, %)*	*Yes (n, %)*
Parental smoking		188 (73.4)	68 (26.6)
Asthma history		255 (99.6)	1 (0.4)
Allergic history		255 (99.6)	1 (0.4)

Values are presented as mean (SD), median (IQR [range]) and n (%). M; male, F; female, SAD; supraglottic airway device, ASA PS; American society of anaesthesiologists physical status, URI; upper respiratory infection, n/a; not applicable. ^a^Range of data: from 0 to 24. ^b^Range of data: from 0 to 20.

### Primary outcome

In our entire cohort, URI scores did not correlate with the atelectasis scores at both time points (after induction or at the end of surgery) (Table 3). In terms of other URI-related medical history, URI onset day was correlated with the B-line score at the at the end of surgery (β = −0.18, p = 0.023). The more recent the onset of URI, the worse the degree of B-line at the end of surgery.

**Table 3:** Multivariable analysis of factors associated with the atelectasis score measured at the post-induction and before-emergence periods using transthoracic lung ultrasonography.

Variables	After induction				At the end of surgery			
	Consolidation		B-line		Consolidation		B-line	
	β	p value	β	p value	β	p value	β	p value
*URI-related*								
URI score^a^	0.13	0.31	0.31	0.098	−0.04	0.762	0.23	0.223
URI onset day^b^	0.00	0.987	−0.15	0.053	−0.07	0.219	−0.18	0.023
URI medication (N vs. Y)	−0.12	0.858	1.22	0.184	0.71	0.292	1.37	0.147
*General characteristics*								
Age	−0.09	< .0001	−0.12	< .0001	−0.07	0.001	−0.08	0.005
Weight	0.14	0.003	0.06	0.377	0.14	0.006	0.13	0.056
Height	−0.02	0.373	0.00	0.95	−0.04	0.124	−0.02	0.626
Sex (F *vs*. M)	0.36	0.59	1.30	0.168	−0.35	0.609	0.81	0.403
Surgery (GS *vs*. urologic)	−0.79	0.153	0.12	0.881	0.20	0.729	−0.10	0.901
Airway (SAD *vs*. intubation)	0.01	0.991	−0.46	0.617	−0.58	0.40	−0.75	0.436
ASA PS (1 *vs.* 2)	0.75	0.064	1.55	0.006	0.07	0.876	1.81	0.002
(1 *vs.* 3)	2.68	0.336	7.11	0.069	1.56	0.585	4.28	0.29
Anaesthesia time	−0.01	0.265	0.00	0.912	−0.00	0.836	0.01	0.474
*Parental information*								
Parental smoking (N vs. Y)	−0.10	0.806	0.44	0.457	−0.52	0.238	0.39	0.526

Values are presented as coefficient; β and p value. URI; upper respiratory infection, N; no, Y; yes, F; female, M; male, GS; general surgery, SAD; supraglottic airway device, ASA PS; American society of anaesthesiologists physical status. ^a^Range of data: from 0 to 24. ^b^Range of data: from 0 to 20.

### Secondary outcome

The age of the patient was associated with the atelectasis scores at both time points; younger children tended to have more B-lines and more consolidation after induction and at the end of surgery. Higher ASA PS was also related to B-lines perioperatively (Table 3).

Based on the above findings, we categorised the children into two groups by age: infants (< 12 months) and toddlers (≥ 12 months). We also divided the children into the following two groups by severity of URI: children with a score of 0 or 1 (no or minimal URI) and those with a score ≥ 2 (mild or moderate URI). In the URI score ≥ 2 group, the scores of B-line finding were higher than those in the URI score 0 or 1 group, but only in the toddlers (Table 4).

**Table 4:** Atelectasis scores (B-line, Consolidation) according to patient’s age after induction and at the end of the surgery periods; URI score < 2 (no or minimal URI) *vs*. ≥ 2 (mild or moderate URI).

Sonographic finding		URI score < 2		URI score ≥ 2	Difference (95% CI)		p value
	*After induction*						
B-line	All age		7.98 (4.13)	9.18 (4.39)		1.20 (0.11—2.30)	0.031
	< 12 months		10.08 (3.58)	11.36 (4.53)		1.28 (-0.54—3.10)	0.172
	≥ 12 months		6.70 (3.93)	8.33 (4.06)		1.63 (0.38—2.87)	0.013
Consolidation	All age		3.09 (2.87)	3.66 (3.06)		0.57 (-0.19—1.33)	0.142
	< 12 months		4.30 (3.10)	4.64 (2.91)		0.34 (-1.10—1.77)	0.641
	≥ 12 months		2.36 (2.47)	3.28 (3.05)		0.93 (0.78—1.77)	0.028
	*At the end of the surgery*						
B-line	All age		8.79 (4.20)	9.71 (4.54)		0.92 (-0.20—2.03)	0.114
	< 12 months		10.49 (4.16)	10.60 (4.49)		0.11 (-1.90—2.11)	0.923
	≥ 12 months		7.76 (3.89)	9.36 (4.54)		1.60 (0.30—2.90)	0.021
Consolidation	All age		3.50 (3.03)	3.57 (2.86)		0.07 (-0.70—0.83)	0.863
	< 12 months		5.00 (3.34)	4.84 (2.61)		-0.16 (-1.64—1.32)	0.834
	≥ 12 months		2.60 (2.42)	3.08 (2.81)		0.48 (-0.33—1.29)	0.241

Values are presented as mean (SD) and mean difference (95% confidence interval).

CI; confidence interval, URI; upper respiratory infection.

PRAEs occurred in 14 of 261 patients (5.4%). The numbers of patients with each symptom were as follows; sustained cough, 5; laryngospasm, 4; bronchospasm, 2; SpO_2_ < 95% in room air, 2 immediately after emergence and 1 in the PACU. The presence of URI symptoms significantly increased the risk of PRAEs (odds ratios [OR] 7.63, 95% confidence interval [CI] 1.67–34.80, p = 0.009). Furthermore, children with higher URI scores were more likely to have PRAEs (OR 1.36, 95% CI 1.10–1.67, p = 0.004). There were no correlations observed between the occurrence of PRAEs and perioperative sonographic atelectasis findings (Table 5).

**Table 5:** Univariate analysis of risk factors for perioperative respiratory adverse events.

		OR (95% CI)	p value
*URI-related*			
URI symptom	no *vs.* yes	7.63 (1.67—34.8)	0.009
URI score^a^		1.36 (1.10—1.67)	0.004
URI onset day^b^		1.05 (0.97—1.14)	0.253
URI medication	no *vs*. yes	2.68 (0.91—7.94)	0.075
*Ultrasonographic finding*			
After induction	consolidation	1.14 (0.97—1.35)	0.118
	B-line	1.05 (0.92—1.20)	0.462
At the end of surgery	consolidation	1.16 (0.99—1.36)	0.060
	B-line	1.12 (0.99—1.26)	0.074
*General characteristics*			
Age (month)		1.00 (0.97—1.03)	0.957
Weight (kg)		1.00 (0.89—1.12)	0.978
Height (cm)		1.01 (0.97—1.06)	0.531
Sex	F *vs.* M	0.54 (0.11—2.57)	0.438
Surgery	GS *vs*. Urologic	0.41 (0.12—1.37)	0.147
Airway device	SAD *vs.* intubation	0.47 (0.06—3.73)	0.477
ASA PS	1 *vs*.2	1.16 (0.40—3.34)	0.782
	1 *vs*.3	5.7 (0.06—560.20)	0.457
Operation time		0.99 (0.97—1.01)	0.425
Anaesthetic time		0.99 (0.97—1.01)	0.336
*Parental information*			
Parental smoking	no *vs.* yes	1.14 (0.35—3.77)	0.827

Values are presented as the odds ratio (95% confidential interval). OR; odds ratio, CI; confidence interval, URI; upper respiratory infection, F; female, M; male, GS; general surgery, SAD; supraglottic airway device, ASA PS; American society of anaesthesiologists physical status. ^a^Range of data: from 0 to 24. ^b^Range of data: from 0 to 20.

Only one child presented with oxygen saturation less than 95% in room air in the PACU. This child received 5 L min^−1^ of oxygen through a shower tent in the ward. He had been diagnosed with bronchiolitis 7 days prior to surgery and was treated from the onset until the day of surgery. The patient did not have any symptoms other than cough (score 2: occasional coughs, and total URI score was 2) on the day of surgery. Postoperative chest radiography showed subsegmental atelectasis in the right lower lobe. He recovered spontaneously the next day and was discharged.

## Discussion

In this prospective observational study, we assessed the correlation between the severity of URI symptoms and anaesthesia-induced atelectasis measured by ultrasonography in children aged 6 months to 6 years. Across the entire age cohort, mild URI was not related to aggravation of anaesthesia-induced atelectasis.

We found no correlation between URI score and atelectasis score in young children overall. One possible explanation for this finding is that the distribution of severity of URI was neither even nor wide; the mean score was 1.43, and the maximum score in this study was only 9 points out of a possible 24. In actual clinical situations, children with higher URI scores are rarely seen in the operating room because elective surgery is postponed if a child has severe URI symptoms. A number of children in our study population presented with higher URI scores and their procedure was rescheduled. Therefore, the patients in this study seem to accurately reflect the actual population of children that we face in the operating room, and we can postulate that minimal or mild URI symptoms are less likely to aggravate perioperative atelectasis to a clinically significant level.

Meanwhile, regardless of the symptoms of URI, there was a clear association between age and atelectasis. We had already excluded babies younger than 6 months because of the greater incidence of perioperative atelectasis in younger children^3,14^, which could obscure any effects of URI. Nevertheless, age remained significantly related to the severity of atelectasis in our result, and younger children were more prone to development of atelectasis after anaesthesia induction as well as at the end of surgery. Accordingly, strategies to reduce atelectasis should be considered and applied for infants and younger toddlers during both induction and maintenance of anaesthesia.

We categorised the children into the following two groups: children with a score of 0 or 1 (no or minimal URI) and those with a score ≥ 2 (mild or moderate URI). In toddlers, the B-lines were significantly more severe in the URI score ≥ 2 group than in the URI score of 0 or 1 group, both after induction and at the end of surgery. B-lines are vertically oriented artefacts which indicate an abnormality in the interstitial or alveolar compartment and can be used to estimate the extent of the altered lung parenchyma, whether it is from extravascular water or inflammation^13^. The number of B-lines correlates with the extent of parenchymal changes on CT^9^. This result can be interpreted that mild current URI have some detrimental effect such as minor atelectasis or infection/inflammation on lower respiratory tracts, and general anaesthesia does not seem to aggravate it.

von Ungern-Sternberg noted that present or recent URI is associated with an increased risk for PRAEs, but the analysis in that study did not consider the severity of URI^2^. Another research group developed the COLDS score, which assesses the **c**urrent signs and symptoms, **o**nset of symptoms, presence of **l**ung disease, airway **d**evices, and **s**urgery, and showed that higher COLDS scores possibly predict PRAEs^15^. However, Lee’s study did not reveal that how each factor would contribute to the occurrence of PRAEs, and the severity of symptoms was categorized as none, mild, and moderate/severe. Our study showed that the incidence of PRAEs was significantly higher in children with only mild symptoms of URI and that severity differences in even mild URI could influence PRAEs.

Diagnosis of atelectasis using lung ultrasound has been widely used recently. It can be easily applied to patients in the operating room and is free of radiation exposure. Moreover, the sensitivity of atelectasis diagnosis is as high as 88%^13^. Ultrasonographic findings are known to highly correlate with the atelectasis volume measured on computed tomography (CT)^16^, and CT yields more accurate diagnoses of atelectasis than chest radiographs^17^. However, clinically significant differences in atelectasis scores using sonography have not been evaluated. Song et al. scanned the same lesions we did and obtained atelectasis scores, but they defined anaesthesia-induced atelectasis to be significant if any region had a consolidation score of  ≥ 2. Another author adopted a completely different scoring system to compare sonographic findings of atelectasis^4^. Therefore, a consensus on the criteria in terms of clinically meaningful scores or findings needs to be established.

There are several limitations of this study. First, any surgical procedures that may affect pulmonary function were excluded; accordingly, children undergoing urologic or lower abdominal procedures were recruited, and the proportion of male children was higher. Second, the validity of the URI scoring system in this population could be questioned. It was introduced by Tailor et al. in 2010 for use in clinical studies to differentiate children with cold symptoms and was based on children aged 2–10 years. According to the authors, its sensitivity is 81.4%, specificity is 61.9%, and accuracy is 73.3%^12^. It is the only objective scoring system available. However, the children enrolled in our study were 6 months to 6 years of age and were generally younger than the children in the original study. Third, airway devices were chosen as routinely used, and SADs were used in more than 85% of the children; accordingly, some cases did not reach the pressure of 30 cmH_2_O because sealing pressure was below 30 cmH_2_O, leading to the probability of incomplete alveolar recruitment. Nevertheless, the use of SADs was not associated with atelectasis in comparison with intubation in the result, and rather reduced the incidence of PRAEs, which was only 5.4% in this study.

In conclusion, children who received anaesthesia in the operating room often had mild URI symptoms, which did not aggravate anaesthesia-induced atelectasis. However, the results of this study emphasised that the risk of PRAEs should always be considered during anaesthetic care of children with even mild URI, and the risk is correlated with the severity of PRAEs.
